# A Case of Ventricular Fibrillation in Asymptomatic COVID-19

**DOI:** 10.7759/cureus.15952

**Published:** 2021-06-27

**Authors:** Amr Abdelradi, Arshad Yekta

**Affiliations:** 1 Internal Medicine, Norwalk Hospital, Norwalk, USA; 2 Cardiology, Norwalk Hospital, Norwalk, USA

**Keywords:** ventricular fibrillation (vf) storm, covid-19, implantable cardioverter-defibrillator, sudden cardiac arrest, acute cardiac injury

## Abstract

In this report, we present a case of ventricular fibrillation (VF) arrest in an asymptomatic coronavirus disease 2019 (COVID-19) patient with no cardiac history and normal cardiac workup. Cardiac involvement in COVID-19 has been described previously in hospitalized patients with severe COVID-19. To our knowledge, this is the first report to describe VF arrest in a patient who was incidentally found to have COVID-19.

## Introduction

The coronavirus disease 2019 (COVID-19) pandemic continues to be a global crisis of epic proportions. As the pandemic unfolds, we are learning more about the extra-pulmonary effects of the virus. Cardiac sequelae of the virus are of particular interest due to the health and economic burdens this could impose on communities worldwide. Multiple reports have documented elevated cardiac biomarkers, arrhythmias, and echocardiographic evidence of cardiac dysfunction in cases of severe COVID-19 infection, which unfortunately confers a poor prognosis. We present a case of out-of-hospital ventricular fibrillation (VF) in a healthy, asymptomatic COVID-19 patient.

## Case presentation

A 40-year-old male with no past medical history was found unconscious by bystanders after he had been out for a recreational jog. Upon the arrival of medical services, the patient was found to be in VF cardiac arrest and required two shocks before the return of spontaneous circulation. The downtime was reported to be about 15 minutes. Per history obtained from family members, the patient had never experienced any cardiac symptoms such as palpitations or chest pain and had no COVID-19 symptoms.

The patient had no significant personal medical history, took no medications, and exercised regularly with no difficulties. Family history was notable for coronary artery disease (CAD) in his brother, which had been diagnosed at age 50, and myocardial infarction (MI) in his father at age 72. There was no family history of sudden cardiac death.

Given the patient’s young age and absence of underlying cardiac issues, the differentials for his VF arrest were broad. Etiologies considered included ischemic heart disease, structural heart diseases such as hypertrophic cardiomyopathy, anomalous origin of coronary arteries, myocarditis, arrhythmogenic right ventricular cardiomyopathy, primary electrical abnormalities such as long QT syndrome, and Brugada syndrome, and non-cardiac etiologies such as a massive pulmonary embolism (PE).

**Figure 1:**
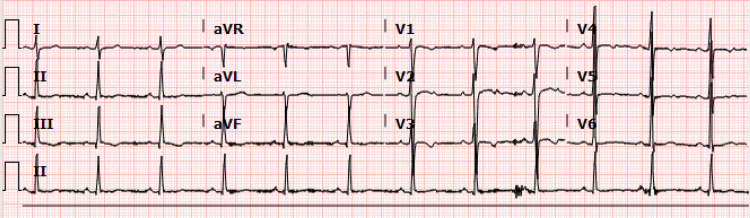
Surface 12-lead electrocardiogram

A basic metabolic panel showed no significant electrolyte abnormalities. The initial troponin-T level was within normal limits. Electrocardiogram (ECG) on admission showed normal sinus rhythm, non-specific T-wave changes in anteroseptal leads, and a QTc of 445 ms (Figure 1). A transthoracic echocardiogram (TTE) showed normal LV systolic and diastolic functions with no valvular pathology or regional wall motion abnormalities. A chest CT with PE protocol showed no significant pulmonary emboli. Per institutional policy, the patient was tested for severe acute respiratory syndrome coronavirus 2 (SARS-CoV-2) via nasopharyngeal swab polymerase chain reaction (PCR) test, which returned positive. A subsequent SARS-CoV-2 antibody test was also positive.

**Figure 2:**
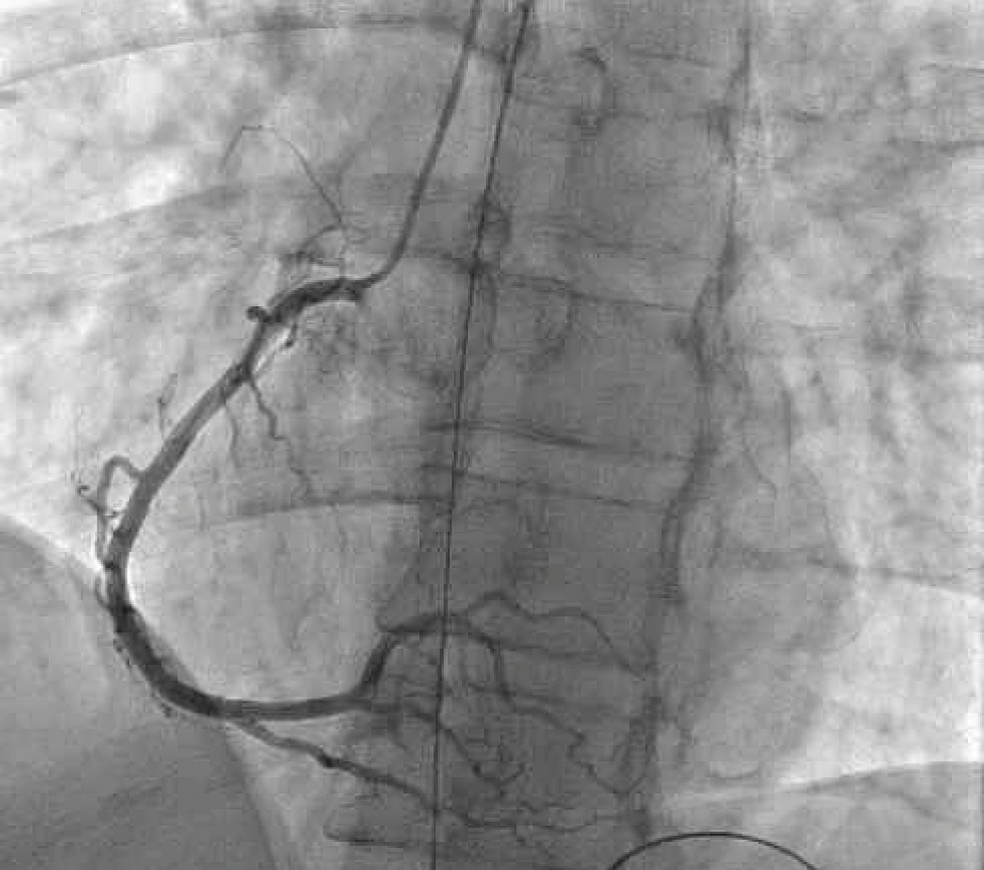
Right coronary angiography showing no significant disease

**Figure 3:**
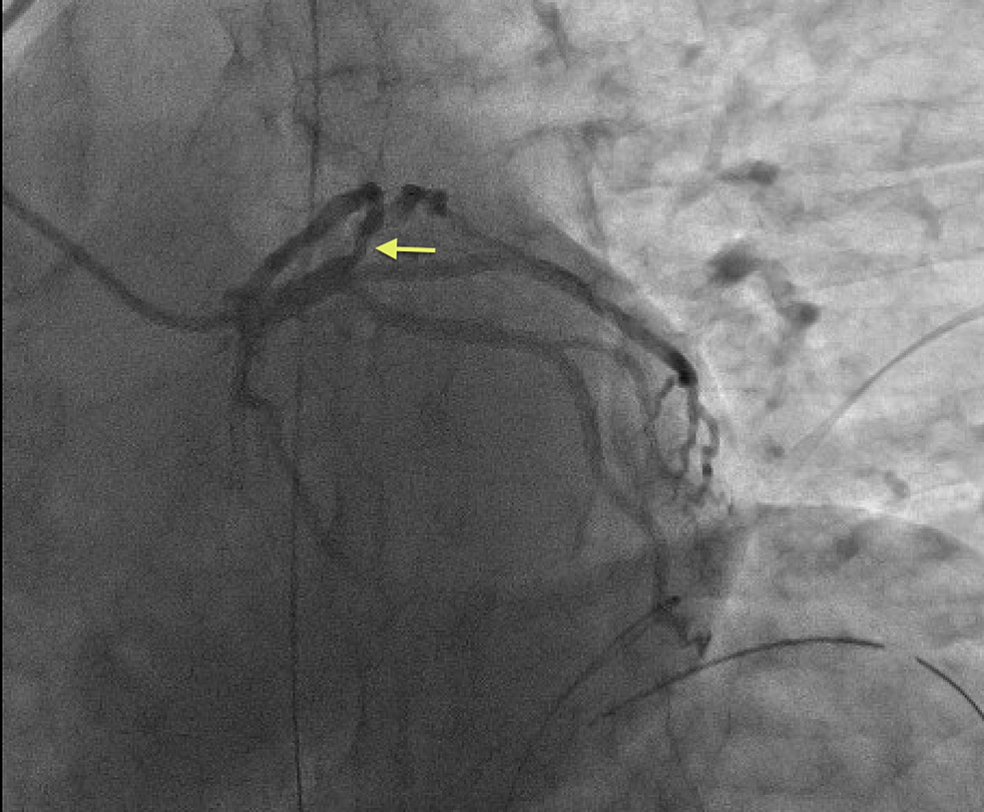
Left coronary angiogram (LAO caudal view) showing mild, non-obstructive proximal LAD lesion (yellow arrow) LAO: left anterior oblique; LAD: left anterior descending artery

The patient was emergently intubated for airway protection and taken to the catheterization lab where coronary angiography showed non-obstructive proximal left anterior descending (LAD) artery disease with 55-60% stenosis and flow distal to the lesion with Thrombolysis in Myocardial Infarction (TIMI) score of 3. Left circumflex and right coronary arteries were disease-free (Figures 2, 3). The left ventriculogram showed normal systolic function with no wall motion abnormalities. The patient was then admitted to the ICU for therapeutic hypothermia. He received convalescent plasma, remdesivir, and dexamethasone for his COVID-19 infection. During his ICU stay, he had one episode of monomorphic ventricular tachycardia (VT) in the setting of agitation and hypoxia that terminated spontaneously in less than three seconds. He continued to improve and was eventually extubated and discharged home.

**Figure 4:**
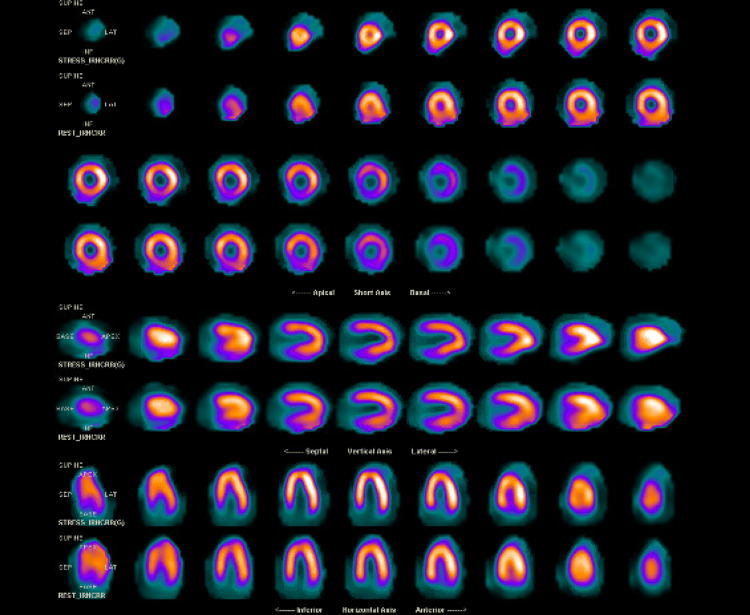
SPECT myocardial perfusion with no evidence of ischemia or scar SPECT: single-photon emission computed tomography

Later on, an outpatient single-photon emission CT (SPECT) pharmacological myocardial perfusion scan showed no anterior ischemia or scar (Figure 4). A subsequent cardiovascular magnetic resonance (CMR) imaging showed normal LV and RV sizes and functions with no late gadolinium enhancement. The patient eventually received a single-chamber VVI implantable cardiac defibrillator (ICD) for secondary prevention of VF. Subsequent outpatient genetic testing with INVITAE® Arrhythmia and Cardiomyopathy Comprehensive Panel (Invitae, San Francisco, CA) revealed no genetic variants known to cause arrhythmia. At the one- and four-month follow-ups, the patient remained asymptomatic and ICD interrogation revealed normal sinus rhythm with no episodes of arrhythmia.

## Discussion

Sudden cardiac arrest (SCA) is a morbid event that can affect even healthy patients with no apparent underlying cardiac disease. One study estimates that 5-10% of SCAs occur in patients with no identifiable structural heart disease [1]. Survivors of SCA should undergo extensive workup to delineate the possible etiology of their arrest.

We discussed the case of a young healthy patient with no underlying cardiac disease who had a VF cardiac arrest in the setting of asymptomatic COVID-19. Our workup, which included TTE, CMR, and coronary angiography, did not reveal any underlying structural abnormality to explain the event. Significant ischemia was also ruled out with the coronary angiogram and the regadenoson myocardial perfusion imaging study. Primary electrical abnormalities such as long QT syndrome and Brugada syndrome were also ruled out based on multiple ECGs. 

Our patient had COVID-19 at the time of his cardiac arrest, which raised the possibility of a COVID-19-related arrhythmia. Although COVID-19 was initially known as primarily a lung disease, multiple reports have documented cardiac, nephrologic, and hematologic involvement in the condition. The exact mechanism by which SARS-CoV-2 affects the heart is not yet fully understood. However, an autopsy study by Lindner et al. has documented the presence of significant viral load in cardiac tissue of 41% of their COVID-19 patients, which may indicate direct viral toxicity as a mechanism of injury [2]. Others cite the concomitant cytokine storm in severely ill COVID-19 patients as a possible mechanism of the multi-organ dysfunction [3]. Moreover, a recent pathologic study by Pellegrini et al. suggests intramyocardial microthrombi as a possible mechanism of cardiac injury, which was found in 64% of their subjects with cardiomyocyte necrosis [4]. Interestingly, these microthrombi had a different composition than coronary thrombi, which may suggest a different pathogenesis.

The clinical manifestations of myocardial injury associated with COVID-19 range from symptoms such as chest pain and palpitations to fulminant cardiogenic shock [5]. Arrhythmias associated with COVID-19 have also been described. According to a multinational survey by Coromilas et al., the most common arrhythmia associated with COVID-19 were atrial arrhythmias (81.8%) whereas VF was the least common, occurring in only 3.1% of patients [6]. Another study by Bhatla et al. describing cardiac arrhythmias in 700 hospitalized COVID-19 patients showed that nine ICU patients had either pulseless electrical activity (PEA) or asystolic cardiac arrest [7]. Both these studies involved hospitalized COVID-19 patients who were severely ill, which may suggest that their cardiac arrests resulted from systemic illness rather than COVID-19 itself.

Our patient had VF in the setting of asymptomatic COVID-19, which was possibly from transient ischemia given the anteroseptal T-wave changes and mild LAD lesion. Genetic testing for channelopathies associated with VF was also negative, thereby making this a less likely etiology.

## Conclusions

We presented a case of VF arrest in a healthy patient who was incidentally found to have asymptomatic COVID-19. Given the ECG and coronary angiographic findings, it is possible that the patient had an episode of transient ischemia that led to the episode. A causal link between COVID-19 and VF cannot be drawn based on this report; however, it still raises the question as to whether there is any association, especially since cardiac involvement is a well-established entity in severe cases of COVID-19 with systemic involvement. Further surveillance of cardiac symptoms in both non-hospitalized and hospitalized patients is required to gain more insight into the cardiac effects of SARS-CoV-2.
